# Surveillance of Cannabis Strains Using Online Data: Observational Study

**DOI:** 10.2196/89897

**Published:** 2026-06-02

**Authors:** Dong Won Yoon, Zefeng Qiu, Hojin Park, Jian Chen, Ce Shang

**Affiliations:** 1 Center for Tobacco Research, The Ohio State University Wexner Medical Center, Columbus, OH, United States; 2 Department of Computer Science and Engineering, The Ohio State University, Columbus, OH, United States; 3 Department of Economics, Chosun University, Gwangju, Republic of Korea; 4 Department of Internal Medicine, Division of Medical Oncology, College of Medicine, The Ohio State University Wexner Medical Center, 3650 Olentangy River Rd, Columbus, OH, 43214, United States, 1 9178688682

**Keywords:** cannabis strains, strain growth trend, cannabis surveillance, online surveilance of cannabis market, marijuana

## Abstract

**Background:**

The flower strains of cannabis are an important attribute that determines product appeal and health impacts. However, there is a lack of surveillance of cannabis strains as the marketplace expands in response to the growing legalization of recreational and medical cannabis.

**Objective:**

This study aimed to analyze cannabis strains and their growth using existing data from user-generated reviews on the Leafly website.

**Methods:**

Using the time stamps of each review, we constructed a dataset of the number of reviews by strain and year over the period of 2010 to 2023. We further conducted trend analyses using Joinpoint regression.

**Results:**

As of December 2023, more than 7000 cannabis strains are available for view or purchase on the website, including more than 6000 that have been reviewed by users or customers. The number of strains with reviews experienced a 13-fold increase from 280 strains in 2010 to 3905 strains in 2023. Strains that are hybrid and contain more than 20% tetrahydrocannabinol experienced the highest growth in review numbers.

**Conclusions:**

The rapid growth of strain numbers poses challenges to cannabis regulation. Policymakers who would like to base regulations on product features need to consider the variability of strains, along with other features such as potency levels and product forms.

## Introduction

The use of cannabis products containing tetrahydrocannabinol (THC) and cannabidiol (CBD) in the United States has been increasing significantly over the past several decades, likely due to the legalization of medical and recreational cannabis by many states [1-3]. In 2021, a total of 19% of the US population, or 52.5 million people, reported using cannabis [4]. While cannabis use for medical purposes, such as pain treatment, could be beneficial, harmful use of cannabis can cause addiction, psychosis, and other severe health consequences [5,6]. Moreover, cannabis use has become more prevalent among teenagers, young adults, and women, making it a public health concern for regulators and the public [7-10].

One critical product feature associated with the benefits and harms of cannabis products is the strain type, which refers to the different varieties of the cannabis plant, each with its own unique set of characteristics, such as potency, flavors, aromas, and effects, shaped by varying chemical profiles, including THC and CBD content [11]. While the therapeutic effects of cannabis products primarily come from CBD, the potency and psychoactive or “high” effects of cannabis primarily come from THC [12]. In addition to CBD and THC, cannabigerol (CBG) is also a chemical commonly listed by dispensaries. Similar to CBD, CBG does not provide a “high” feeling or psychoactive effect but may have some therapeutic benefits [13].

Recent data have shown that, as a growing number of states legalize both medical and recreational cannabis use, the potency or THC levels of cannabis products in the marketplace have risen drastically [14,15]. This is likely driven by the increasing variety of hybrid strains that provide a wide range of THC-to-CBD ratios and CBG levels [16-19]. Therefore, it is important to track the dynamic trends of cannabis strains that facilitate increases in product potency.

Moreover, as the commercialization of cannabis expands in the United States, it has resulted in the proliferation of many commercial cultivators and the growth of strain varieties. In that respect, tracking the life cycles of cannabis strains and their popularity can shed light on the evolving marketplace and consumer preferences, which provide important information for policymakers.

Publicly accessible platforms for cannabis dispensaries and product information, such as Weedmaps.com and Leafly.com, are valuable sources for tracking cannabis strains and their evolution [20]. Recent research has scraped Weedmaps.com to study attributes such as product types and flavors [20]. However, no studies have examined strains using data from Leafly.com, an alternative information hub to Weedmaps. In addition, Leafly.com provides ongoing reviews of cannabis strains with time stamps that allow researchers to assess the emergence of strains and their popularity.

In this study, we examined the emergence of all cannabis strains listed on Leafly.com as of December 2023. We hypothesized that the number of unique strains, especially the number of high-potency strains, has increased significantly over time. This study aims to provide valuable insights into how cannabis strains have evolved over time and to offer insights for stakeholders and policymakers who are considering regulating cannabis strains.

## Methods

### Study Design

This is an observational surveillance study that conducted secondary data analyses of existing data on cannabis features and consumer reviews, including strain information.

### Data Source

We analyzed a previously collected dataset of 7037 cannabis strains with review information spanning from 2010 to December 2023 from Leafly.com, a leading platform for providing detailed information on cannabis products and connecting users with legal, licensed retailers [21]. While Weedmap.com is also a popular source, Leafly.com is better suited for obtaining strain information in a systematic way based on assessment [22]. Specifically, all strain reviews on Leafly.com show time stamps indicating when each review was posted, which is critical for building long-term trend and panel data (strain review×time). Other strain-related information and the detailed data collection methods of this dataset are published online and are available in an online repository [22]. Specifically, the data collection results were verified using large language models. All listed strains were included without exclusion, and no duplications were found during data processing. The data can be accessed online with processing codes for future replication.

### Measures

#### Number of Strain Reviews

The primary outcome variables of this study are the number of reviews per year across all strains and per strain from 2010 to 2023. We also constructed a stock variable representing the total number of reviews per strain as of December 2023. These variables reflect the popularity of each strain [23,24].

#### Strain Names

The strain names (eg, Blue Moon) and their strain or plant types (ie, sativa, indica, or hybrid as a hybrid of the two) are provided by Leafly.com based on information from manufacturers. Regarding strain types, sativa refers to cannabis plants originally from India, and indica refers to plants originally from Central Asia [17]. These names are based on a snapshot in 2023 at the time of data collection.

#### Strain Feature Classifications

The strain feature classifications are available for key features listed by Leafly.com, including THC, CBD, and CBG levels as percentages. These classifications are provided by Leafly.com based on information from manufacturers. We consider strains that contain a potency level of 20% or above as high-potency strains [25]. The strain features are based on a snapshot in 2023 at the time of data collection.

#### Number of Unique Strains With Reviews

To track cannabis strains that may have expanded during the last decade, we constructed the number of unique strains with reviews per year for the period of 2010 to 2023. This measure reflects the availability of strain types over time.

#### Number of Unique Strains With the First Review

To track the emergence of new strains over time, we constructed the number of strains with the first review for each year from 2010 to 2023. While there may be gaps between when the strain was first launched into the market and when it received its first review on Leafly.com, the number of strains with the first review is still a useful measure for capturing the emergence of new strains over time.

#### Compound Annual Growth Rate in Review Numbers

We calculated the compound annual growth rate (CAGR) for the entire period (2010‐2023) for each individual strain and for the overall sample. CAGR is ideal for analyzing long-term, consistent growth over multiple years [26-28].

### Data Analyses

#### Descriptive Statistics

First, we present the descriptive statistics of the number of strains and the number of reviews per strain using snapshot data collected in 2023. We further presented the names of strains that received at least 1000 reviews, along with the number of reviews, in a frequency chart.

#### Trend Analysis

We first plot the number of reviews per year over the period 2010 to 2023 for the following 3 groups: all reviewed strains, strains with at least 1000 reviews, and strains with fewer than 60 reviews. The strain popularity was categorized using the following criteria: strains with at least 1000 reviews as of December 2023 were considered popular, and strains with fewer than 60 reviews were considered least popular. These cutoffs were determined based on the distribution of review numbers (mean number of reviews per strain 59.96, SD 277.48).

We further conducted Joinpoint regressions to identify trend segments and assess annual percent changes in review measures and their significance levels (*P*<.05).

#### Change and Variability Analysis

To assess review growth for the top 10 strains with the highest number of reviews as of December 2023, we calculated both the CAGR to represent a steady growth rate over the period. CAGR reflects total growth from start to end (2010‐2023), while the average annual change highlights yearly fluctuations. CAGR is ideal for analyzing long-term, consistent growth over multiple years, but for single-year changes, simple arithmetic change is usually sufficient [26-28]. The formula of CAGR is following.


$$CAGR\left(t_{0},t_{n}\right)\text{=}{\left(\frac{V\left(t_{n}\right)}{V\left(t_{0}\right)}\right)}^{\frac{1}{t_{n}\text{ - }t_{0}}}\text{ - }1$$

Where $V\left(t_{0}\right)$ is the initial value, $V\left(t_{n}\right)$ is the end value, and $t_{n}\text{ - }t_{0}$ is the number of years.

**Figure 1.**
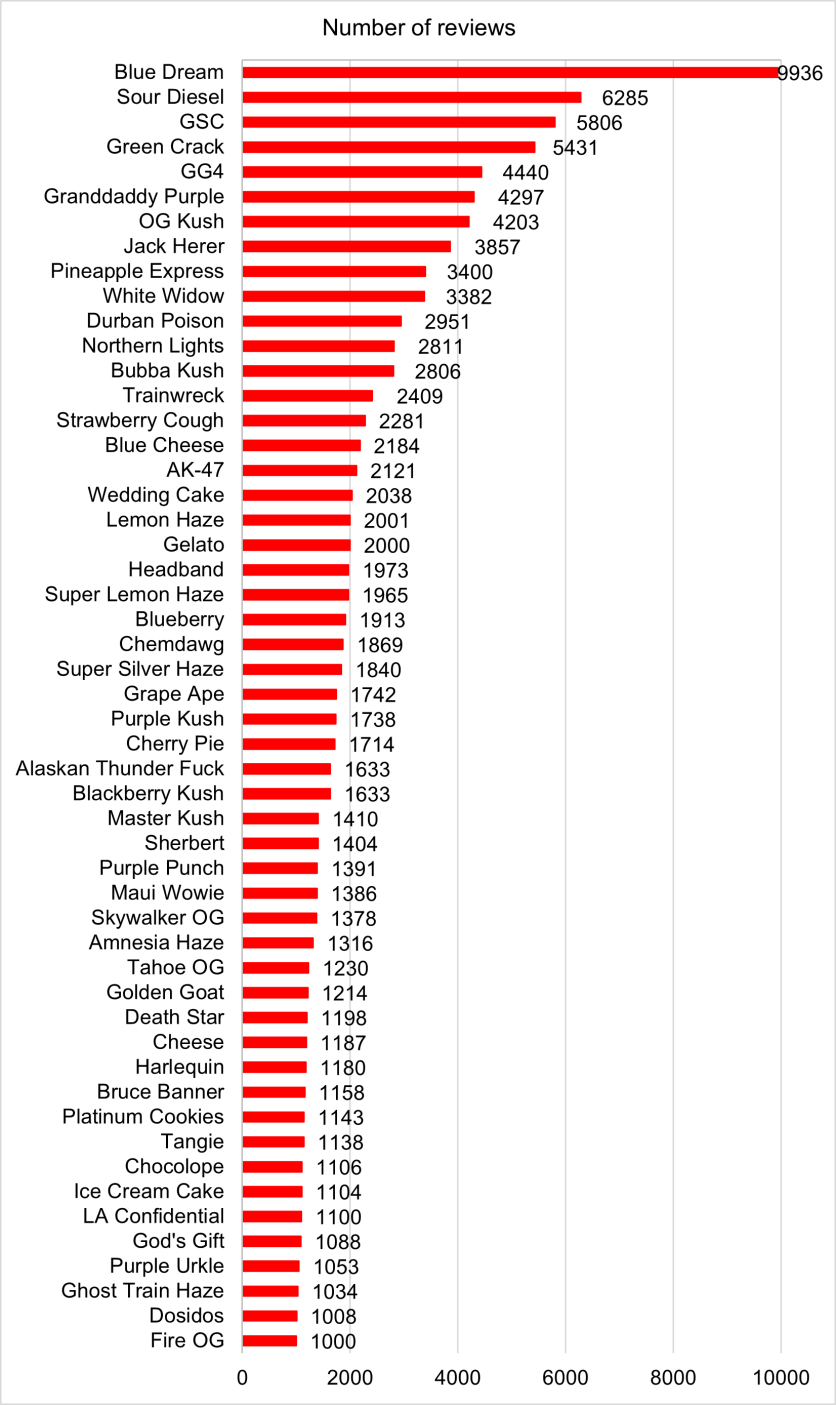
Ranking of review numbers for strains with ≥1000 reviews. GG4: Glue Original Glue; GSC: Girl Scout Cookies.

We also calculated the CAGR for 52 strains that had at least 1000 reviews as of December 2023, as well as for the 6111 strains with reviews (Figure 1). All analyses were conducted using Python (Python Software Foundation) [29], R (R Foundation for Statistical Computing) [30], and Joinpoint (National Cancer Institute) [31].

### Ethical Considerations

This manuscript did not involve human participants and was based on publicly available datasets. The Ohio State University’s institutional review board (2022B0032) has approved this study.

## Results

### Descriptive Statistics

Table 1 presents the number of strains and the number of reviews per strain and further provides summary statistics for strains that have received any reviews (>0). Out of the total 7037 strains, 926 (13.2%) strains received no reviews, 6111 (86.8%) strains received at least one review, 4532 (64.4%) strains had between 1 and 30 reviews, 1579 (22.4%) strains had more than 30 reviews, and 52 (0.7%) strains garnered more than 1000 reviews. For the 6111 strains with at least one review, the mean number of reviews per strain was 59.96 (SD 277.48; range 1-9936). After ranking strains by number of reviews, the lower 25th percentile had 3 reviews, the 50th percentile had 9 reviews, and the upper 25th percentile (75th percentile) had 31 reviews. This table demonstrates a highly uneven distribution of consumer reviews, with 52 strains receiving more than 1000 reviews, while strains at the 75th percentile of the number of reviews receive merely 31 reviews. This pattern suggests that a small portion of strains receive a relatively large number of reviews.

**Table 1.** The number of strains and reviews as of December 2023^a^.

Number of reviews per strain	Strains (n=7037), n (%)
0	926 (13.2)
1‐10	3078 (43.7)
11‐20	976 (13.9)
21‐30	478 (6.8)
31‐40	300 (4.3)
41‐50	204 (2.9)
51‐60	156 (2.2)
61‐70	105 (1.5)
71‐80	84 (1.2)
81‐90	75 (1.1)
91‐100	63 (0.9)
101‐337	399 (5.7)
338‐999	141 (2)
≥1000	52 (0.74)

a For strains with reviews (n=6111), the mean number of reviews per strain was 59.96 (SD 277.48; range 1-9936). The 25th, 50th, and 75th percentiles were 3, 9, and 31 reviews, respectively.

“Blue Dream (THC 21%, CBD 0%; hybrid)” was the standout leader with 9936 reviews, followed by “Sour Diesel (THC 19%, CBD 0%; hybrid)” with 6285 reviews, “Girl Scout Cookies or GSC (THC 25%, CBG 1%; hybrid)” with 5806 reviews, “Green Crack (THC 17%, CBG 1%; sativa)” with 5431 reviews, “Glue Original Glue or GG4 (THC 20%, CBD 0%; hybrid)” with 4440 reviews, “Granddaddy Purple (THC 17%, CBD 0%; indica)” with 4297 reviews, “OG Kush (THC 25%, CBD 0%; hybrid)” with 4203 reviews, and “Jack Herer (THC 18%, CBG 1%; sativa)” with 3857 reviews. These top 10 most reviewed strains include 7 hybrid strains, 2 sativa strains, and 1 indica strain. This ranking is in line with the overall distribution of these types, where hybrid strains significantly outnumber sativa and indica strains [32].

### Trend Analysis of Strain Reviews

**Figure 2.**
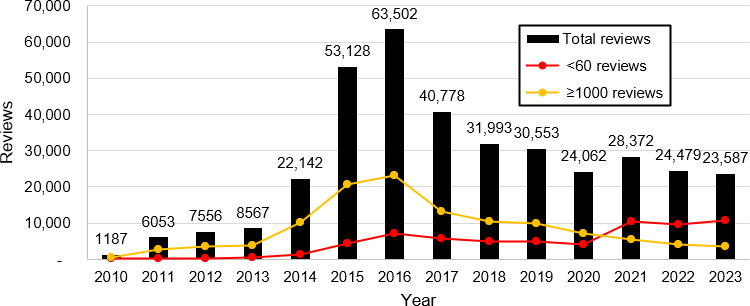
Number of reviews per year (2010‐2023), stratified by total reviews and review count.

Figure 2 shows the number of reviews per year across all strains, as well as by strain popularity. The definition of popularity is described in the Methods section (<60 reviews are considered least popular, and 1000 or more reviewers are considered the most popular). Figure 2 also explicitly presents the total number of reviews over time. The trend for the total number of reviews across all strains shows 2 segments based on Joinpoint analysis. Between 2010 and 2015, the number of reviews was increasing over time, with an average percent change (APC) of 96.68% (*P*<.05). Starting in 2015, the number of reviews decreased, with an APC of −10.07% (*P*<.05).

The number of reviews per strain per year allowed us to identify the most reviewed strain over time (Table 2). In 2010, the most reviewed strain was Sour Diesel (THC 19%, CBD 0%; hybrid). From 2011 to 2019, Blue Dream (THC 21%, CBD 0%; hybrid) was the most reviewed strain. From 2020 to 2023, Wedding Cake (THC 24%, CBG 1%; hybrid) was the most reviewed strain.

**Table 2.** The most reviewed strain by year (2010‐2023).

Strain name and year	Reviews, n
Sour Diesel	
2010	50
Blue Dream	
2011	348
2012	340
2013	441
2014	1209
2015	2038
2016	2008
2017	916
2018	773
2019	757
Wedding Cake	
2020	438
2021	408
2022	277
2023	237

**Figure 3.**
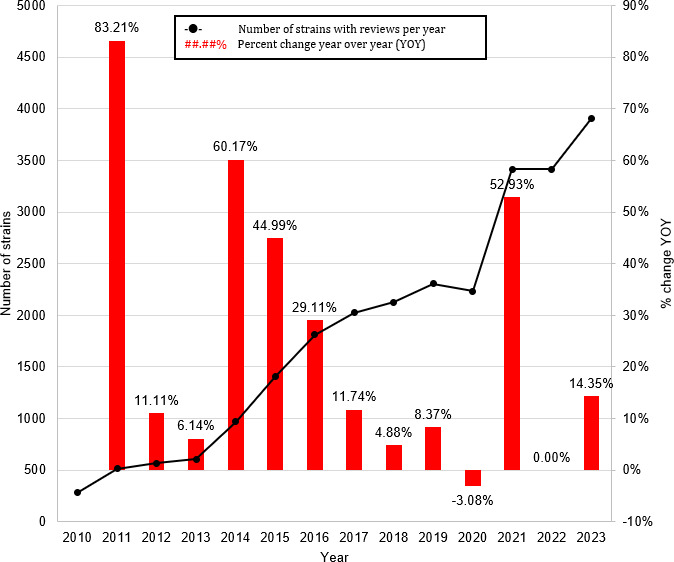
Number of unique strains with reviews per year.

Figure 3 shows the number of unique strains with reviews per year and indicates a steady increase in the number of strains reviewed annually from 2010 (280 strains) to 2023 (3905 strains), with notable growth over time. The increasing trend for the number of unique strains with reviews per year shows 2 segments based on Joinpoint analysis. The APC was 33.09% (*P*<.05) between 2010 and 2016 and 11.76% (*P*<.05) between 2016 and 2023.

Table 3 reports the number of unique strains that received their first review in each year. Between 2010 and 2020, the number of strains receiving their first review each year ranged from 81 to 444. There was a dramatic one-time increase in newly reviewed strains from 345 in 2020 to 1420 in 2021. The Joinpoint analysis identified one increasing trend in the number of unique strains receiving their first reviews, with an APC of 12.99% (*P*<.05).

**Table 3.** Number of unique strains receiving their first review.

Year	Strains, n
2010	280
2011	268
2012	112
2013	81
2014	316
2015	389
2016	444
2017	347
2018	245
2019	405
2020	345
2021	1420
2022	564
2023	873

All 10 top-reviewed strains as of December 2023 experienced higher than 6% growth from 2010 to 2023 (Table 4). Pineapple Express (THC 20%, CBG 1%; hybrid) recorded the highest CAGR growth rate (28.10%), followed by Girl Scout Cookies or GSC (THC 25%, CBG 1%; hybrid) with a growth rate of 24.90%, Glue Original Glue or GG4 (THC 20%, CBD 0%; hybrid) with a growth rate of 11.76%, Blue Dream (THC 21%, CBD 0%; hybrid) with a growth rate of 11.21%, Granddaddy Purple (THC 17%, CBD 0%; indica) with a growth rate of 11.18%, White Widow (THC 15%, CBG 1%; hybrid) with a growth rate of 10.33%, OG Kush (THC 25%, CBD 0%; hybrid) with a growth rate of 9.85%, Jack Herer (THC 18%, CBG 1%; sativa) with a growth rate of 9.17%, Green Crack (THC 17%, CBG 1%; sativa) with a growth rate of 9.02%, and Sour Diesel (THC 19%, CBD 0%; hybrid) with a growth rate of 6.18%. The top 4 strains and out of 10 most reviewed products are high-potency strains with a THC level of 20% or above.

**Table 4.** Growth rates between 2010 to 2023 for the top 10 most reviewed strains as of December 2023.

Strain	Growth rate (%)
Pineapple Express	28.10
Girl Scout Cookies	24.90
Glue Original Glue	11.76
Blue Dream	11.21
Granddaddy Purple	11.18
White Widow	10.33
OG Kush	9.85
Jack Herer	9.17
Green Crack	9.02
Sour Diesel	6.18

### Additional Results to Complement Main Findings

**Figure 4.**
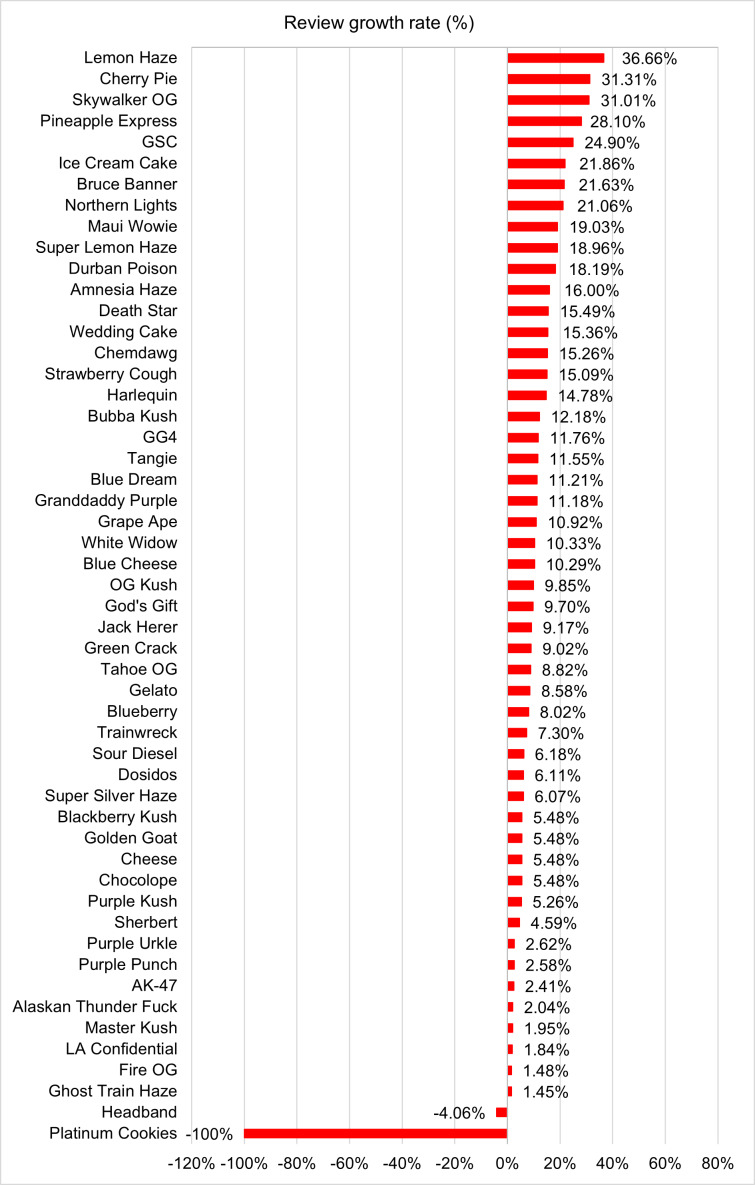
Growth rate of reviews for the top 52 most reviewed strains (reviews ≥1000) as of December 2023. GG4: Glue Original Glue; GSC: Girl Scout Cookies.

Figure 1 demonstrates the names of cannabis strains with more than 1000 reviews, showcasing the most popular ones. Multimedia Appendix 1 plots strain types in a word cloud. We also examined the CAGR for the 52 strains with 1000 or more reviews and ranked them based on their growth rates (Figure 4).

## Discussion

### Principal Findings

As of December 2023, more than 7000 cannabis strains are available for view or purchase on the website, including more than 6000 that have been reviewed by users or customers. Between 2010 and 2023, the number of strains with reviews experienced a 13-fold increase from 280 strains in 2010 to 3905 strains in 2023. In addition, prior to 2020, there were 81 to 400 unique strains receiving their first reviews every year, likely marking their initial launch in the marketplace. This number increased to more than 800 after 2020, suggesting rapid expansion in strain varieties. We further identified the top-reviewed strains and strains that experienced significant growth during 2010 to 2023. The results show that the most reviewed strains have the following features: THC levels greater than 20%, hybrid types, and low or no CBD or CBG in the product.

### Implications and Comparison With Prior Work

As the marketplace of cannabis expands due to increasing legalization of cannabis in the United States, the surveillance of the marketplace lags [20]. In particular, cannabis strains present different combinations of THC and CBD or CBG levels, thereby dictating the harm and therapeutic potential of the flowers and their derivatives [33,34]. Cannabis strains are further associated with consumer perception and use [35-39]. Yet, the variability and evolution of cannabis strains in the United States are seldom studied. Our study innovatively uses publicly available data published by Leafly.com to trace and track the evolution of cannabis strains, filling the data gap. We found that top-reviewed and fast-growing strains are high-potency strains, which coincide with the increasing recreational use of cannabis and the growing potency of cannabis products that have been documented in prior studies [14,33,34].

Our findings add to the growing evidence that cannabis legalization contributes to the availability of products [14]. Our trend analysis revealed that the number of consumer reviews of cannabis strains increased rapidly between 2010 to 2016, coinciding with the legalization of medical and recreational cannabis in populous states (eg, New York legalized medical cannabis in 2014 and California legalized recreational cannabis in 2016) [40,41]. Our observation of the expansion in unique strains also corroborates with Leafly.com’s own documentation that new strains are being launched constantly, further coinciding with increases in cannabis use prevalence and sales [14,32]. The abundance of different strain variabilities calls for more in-depth surveillance and monitoring systems to understand their features (eg, CBD and THC levels, flavors, effects, etc) and appeals, which are critical for both consumers and regulators [33,34].

Our findings demonstrate the challenges of regulating cannabis, as product features constantly change. The increasing number of unique strains is likely driven by the high-potency varieties [14]. This is not surprising, as the market size of recreational or adult cannabis use is expected to far exceed the market size of medical use [14]. A growing number of studies have documented that high-potency products are increasingly available in the US market along with increased use prevalence of these products [14,33,34]. The forms and delivery modes of cannabis also expanded—many cannabis products sold in the market are inhalable vape cartridges with high THC contents, which did not exist a decade ago [14]. Our findings show that cannabis strain is another attribute that has undergone significant diversification to facilitate the growth of high-potency products and its impacts on the mode of consumption (eg, whether for medical or recreational purposes) and public health need to be studied in future research.

### Limitations

This study has some limitations. First, we used data from only one of the 2 prominent cannabis information websites (Leafly.com and Weedmaps.com). Second, while review numbers and their first review dates may respectively reflect popularity and launching time, these measures may not be accurate. Some strains may have accumulated a high number of reviews due to their prolonged presence on the platform, while others may have fewer reviews simply because they were listed for a shorter period. Furthermore, the number of reviews is by unique accounts instead of identifiable individuals, which may bias the data. Third, the study uses online review counts as a proxy for strain popularity, which is subject to marketing influences and other online review biases. Finally, information on strain type (hybrid, sativa, and indica) and features (THC, CBD, and CBG) was obtained from the Leafly platform at the time of data collection. These values were referenced directly from the platform and are not independently verified, standardized, or analytically evaluated within this study. Nevertheless, to our knowledge, this study is the first to analyze cannabis strain reviews on a website using review frequencies to track popular strains and changes in their popularity over time. Future studies are needed to address these limitations.

### Conclusions

In summary, the growth of unique cannabis strains, especially high-potency hybrid strains, suggests that cannabis strains may be used to facilitate recreational cannabis use and the use of high-potency products. The growing strain varieties further attract consumers with tailored taste and desired potency levels. Policymakers who would like to regulate high-potency products may consider regulating strain varieties and strains with high potency by issuing product standards to cap the THC levels in strains or limiting the manufacturing and sales of high-potency strains.

## Supplementary material

10.2196/89897 Multimedia Appendix 1 Word cloud of all reviewed strains weighted by the number of reviews as of December 2023.

## Abbreviations

APC: average percent change
CAGR: compound annual growth rate
CBD: cannabidiol
CBG: cannabigerol
THC: tetrahydrocannabinol
