# Supplementary figures and images for: Surveillance of Cannabis Strains Using Online Data: Observational Study
Source: JMIR Form Res. 2026 Jun 2;10:e89897. doi: 10.2196/89897 (PMC13229463; doi:10.2196/89897)

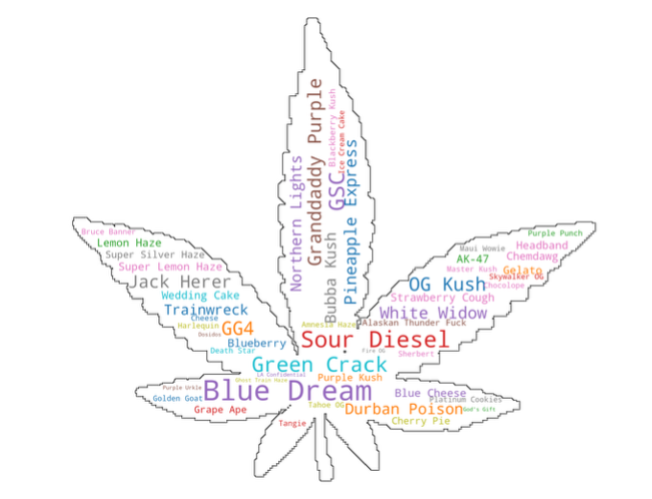

Supplement: Multimedia Appendix 1 [file formative-v10-e89897-s001.png]
